# Proposed Modifiable Scoring Criteria for Studies Included in Meta-Analyses to Reduce Measurement Bias

**DOI:** 10.7759/cureus.84099

**Published:** 2025-05-14

**Authors:** Gabriel Vizgan, Bernard von Simson, Joey Nicholson, Gabrielle Gold-von Simson

**Affiliations:** 1 Urology, State University of New York (SUNY) Downstate Health Sciences University, New York, USA; 2 Economics and Global Affairs, New York University, New York, USA; 3 Statistics, New York University (NYU) Grossman School of Medicine NYU Langone Health, New York, USA; 4 Pediatrics, New York University (NYU) Grossman School of Medicine NYU Langone Health, New York, USA

**Keywords:** bias identification, meta-analysis, novel scoring system, systemic review, validity assessment

## Abstract

Due to inconsistencies in literature reporting, there is potential for bias in meta-regression calculations used in systematic reviews and meta-analyses. To address this, we propose a novel modifiable point-based scoring system, highlighting to readers the scientific weight of included studies relative to the conclusion of the review’s authors. Such a scoring system will have the added benefit of also highlighting data points that are underrepresented. This system is ideal for synthesizing reviews on pathologies poorly represented in the literature. The scoring system described will decrease bias and enable impactful interpretation to ultimately lead to standard practice adaptation.

## Editorial

Introduction

Meta-analyses are arguably the most important form of scientific study. They aggregate data in a way that is clinically relevant and easy to decipher, which can lead to the adoption of clinical guidelines [1]. However, they can also introduce measurement bias [2]. To reduce bias in meta-analyses, guidelines have been put in place to ensure transparency, reproducibility, and generalizability [3]. Even with rigorous guidelines, though, there remains significant potential for bias in meta-regression calculations. This is partly because there is no standardized reporting protocol set across the various publishing bodies, such as a consistent set of data points for each respective topic, leading to inconsistencies in reporting that can impact the data available from which to draw inferences. 

Reporting inconsistencies can result in papers with greater scientific weight being aggregated with others that barely meet the inclusion criteria, skewing the results and altering readers’ perception of the meta-analysis study’s overall validity. Readers are unaware of the scientific value of the individual papers that make up the aggregation, or what metrics came from which paper. As it stands, missing data that cannot be obtained from the study authors remains hidden behind large n values with citations. To solve this, we suggest a modifiable scoring criterion to evaluate the contributive value of each publication included in a meta-analysis, relative to the reviewer’s intended purpose. 

This modifiable scoring system was first conceived and implemented by Vizgan et al. in their systematic review on the surgical outcomes of ketamine-induced uropathy cases [4]. At the time of their study, existing literature had been sparse, and there was no consensus on how to report on the disease complex or its post-surgical measures of success. As such, the authors struggled to aggregate the data in any meaningful way. To combat this, they used the described scoring criteria to portray to readers their initial intentions in conducting the study, the strengths and weaknesses of the existing data, and areas for future research. 

Our scoring differs from Cochrane’s risk-of-bias (RoB) 2 reporting guidelines in that it does not attempt to assess studies for their internal validity [5]. Instead, the proposed scoring criteria are meant to address the limitations of the Meta-analyses of Observational Studies (MOOSE) guidelines by reporting on the heterogeneity and quality of the data disclosed in studies included in meta-analyses [6]. In particular, these scoring criteria will establish a standardized means of addressing the MOOSE requirement of “Assessment of quality of included studies”.

Modifiable scoring criteria 

Desired data should be established before reading any of the studies to add credibility to the review procedure. Meta-analyses consider data typology for statistical analysis. With continuous data (age, respiratory rate, etc.), means and standard deviations are generally utilized. For dichotomous data (yes/no), event rates or proportions are described. Ordinal data (severity scores, Likert scales) may utilize cumulative odds ratios, whereas nominal data (medications, region) more often utilize risk or odds ratios. In addition, time-to-event data will typically use hazard ratios or survival curves. Understanding and correctly categorizing the data is necessary to properly analyze the various studies. Oftentimes, the appropriate data is underreported, and therefore, the proposed scoring criteria are useful.

As depicted in the example in Table 1, the proposed scoring table consists of a list of all such data points along the horizontal axis, and each respective paper to be included in the review is represented on the vertical axis. A publication will receive one point for each data point (i.e., respiratory rate) with an assigned numerical value. For each non-numerical or qualitative data point mentioned (i.e., “patient showed improvement in respiratory distress”), half a point will be allotted. Finally, for each data point a publication does not include in its body, but the author of the meta-analysis wishes to study, it will receive a zero. If data on the scoring chart is not relevant to a specific paper (i.e., postoperative values in a non-surgical case report), an N/A will be awarded for that datum. The authors will then compute the average scores for each paper as the sum of all data points divided by the number of applicable data points (excluding those that received an N/A). The authors will also calculate the average scores of the individual data points using the same method. 

**Table 1:** Sample criteria scoring table using three papers and four data points

	Paper 1	Paper 2	Paper 3	Score
Criterion 1	1	1	1	100%
Criterion 2	0	0.5	1	50%
Preoperative Criterion 3	N/A	1	0	50%
Postoperative Criterion 3	N/A	0.5	1	60%
Criterion 4	0	N/A	0	0%
Score	33%	75%	60%	

From Table 1, one can see that Paper 2 provided the greatest number of data points that were considered relevant to the conclusion of the meta-analysis. Researchers or students may choose to read this paper, or any other with similarly high scores, to get a better understanding of the studied condition. Furthermore, while Criterion 1 was reported on in every included source, Criterion 4 was not included in any of them. This may be valuable information to researchers, as it points to gaps in medical knowledge and areas of future focus. Moreover, it gives insight into the thought process of the meta-analysis authors and documents an aspect of their research methodology that may not have otherwise been published. 

Such a table could become quite large depending on the number of papers included in the systematic review and the number of studied data points. Therefore, to make publication more manageable, the authors of the systematic review or meta-analysis should instead publish a summary table, as shown in Table 2, using the aforementioned criteria. The summary table contains two distinct sections; the left side is composed of two columns listing all of the studied data points and their corresponding average scores on the right. The right side of the table is dedicated to the included studies, with a list of studies on the left and their average score to their right. At the bottom of both segments of the table is an average score.

**Table 2:** Proposed criteria scoring table for journal publication using the data in Table 1

Criteria/Paper	Score
Criterion 1	100%
Criterion 2	50%
Preoperative Criterion 3	50%
Postoperative Criterion 3	60%
Criterion 4	0%
Average	57%
Paper 1	33%
Paper 2	75%
Paper 3	60%
Average	62%

Discussion 

The utility of this novel scoring system is its ability to present to the reader an aggregate of the data in a way that is representative of its weight in the literature. This system is particularly useful when the data available is limited and difficult to collate in a methodological and categorical manner. Through this scoring system, should a reader identify that the majority of the included studies have high scores, implying they include information on most of the data points the review’s authors wished to study, then it stands to reason that any conclusion drawn from the data aggregation is representative of the literature and may further validate the findings of their systemic review or meta-analysis. On the other hand, if most studies or data points have a relatively low score, then it may be inferred that the meta-regressions resulted are potentially skewed, inaccurate, or non-representative of the greater population, regardless of high n values associated with each data point.

Currently, collative reviews of this nature tend to present all of their data in either the paper’s body or in appendices. However, this matter of data presentation can be unwieldy and difficult for readers to draw their own inferences from. In particular, the source of this data and its representative weight in the literature are not made easily apparent and are hidden behind "n" numbers and citations. These scoring criteria would thereby assist in making any internal or external bias more apparent to readers.

Furthermore, the proposed scoring method could provide new inclusion criteria by which authors can judge whether or not to include studies in their reviews based on the score that the paper receives. No journal requires specific data points to be reported so long as the authors can justify their conclusion; the adoption of these scoring criteria may encourage future authors to publish data they deem irrelevant for future review studies to utilize.

It is worth noting that a paper receiving a low score does not imply it is poorly written, nor should it reflect badly on its authors. This method of scoring is solely intended to imply a study’s usefulness in reaching the conclusion of the systematic review or meta-analysis. It is also important to recognize that this method of scoring is completely customizable to the needs of the review author.

Conclusion 

At present, there is no standardized scoring system to assess the scientific value of studies included in a meta-analysis. The scoring system devised and described here can decrease bias and enable more impactful interpretation to ultimately lead to more informed recommendations and guideline creation. 
